# A comprehensive transcriptome data of normal and *Nosema ceranae*-stressed midguts of *Apis mellifera ligustica* workers

**DOI:** 10.1016/j.dib.2019.104349

**Published:** 2019-08-22

**Authors:** Huazhi Chen, Yu Du, Cuiling Xiong, Yanzhen Zheng, Dafu Chen, Rui Guo

**Affiliations:** College of Bee Science, Fujian Agriculture and Forestry University, Fuzhou 350002, China

**Keywords:** *Apis mellifera ligustica*, *Nosema ceranae*, Midgut, Transcriptome

## Abstract

Honeybees are pivotal pollinators of crops and wild flora, and of great importance in supporting critical ecosystem balance. *Nosema ceranae*, a unicellular fungal parasite that infects midgut epithelial cells of honeybees, can dramatically reduce honeybee population and productivity. Here, midguts of *Apis mellifera ligustica* workers at 7 d and 10 d post inoculation (dpi) with sucrose solution (Ac7CK and Ac10CK) and midguts at 7 dpi and 10 dpi with sucrose solution containing *N. ceranae* spores (Ac7T and Ac10T) were sequenced using strand-specific cDNA library construction and next-generation sequencing. A total of 1956129858 raw reads were gained in this article, and after quality control, 1946489304 high-quality clean reads with a mean Q30 of 93.82% were obtained. The rRNA-removed clean reads were then aligned to the reference genome of *Apis mellifera* with TopHat2. For more insight please see “Genome-wide identification of long non-coding RNAs and their regulatory networks involved in *Apis mellifera ligustica* response to *Nosema ceranae* infection” [1]. Raw data were deposited in NCBI Sequence Read Archive (SRA) database under the BioProject number PRJNA406998. These data can be used for comparative analysis to identify differentially expressed coding RNAs and non-coding RNAs involved in *A. m. ligustica* responses to *N. ceranae* stress, and for investigation of molecular mechanisms regulating host *N. ceranae* -response.

**Table:** Specifications table

Subject	*Biology*
Specific subject area	*Transcriptomics*
Type of data	*Table, Figure*
How data were acquired	*Illumina Hiseq^TM^ 4000*
Data format	*Raw sequences (FASTQ) and processed data (FASTA)*
Experimental factors	*Normal and Nosema ceranae stressed midguts of Apis mellifera ligustica workers*
Experimental features	*Midgut samples in control groups were collected from A. m. ligustica workers inoculated with sterile sucrose solution, while midgut samples in treatment groups were collected from workers inoculated with sterile sucrose solution containing N. ceranae spores. Total RNA of control and treatment groups were isolated followed by strand-specific cDNA library construction and deep sequencing.*
Data source location	*College of Bee Science, Fujian Agriculture and Forestry University, Fuzhou, China*
Data accessibility	*Raw data of RNA-seq are available on Sequence Read Archive (SRA) database and connected to BioProject PRJNA406998.*
Related research article	*R. Guo, H.Z Chen, Y. Du, D.D. Zhou, S.H. Geng, H.P. Wang, Z.W. Zhu, C.Y. Shi, J.Q. Wan, C.L. Xiong, Y.Z. Zheng, D.F. Chen. Genome-wide identification of long non-coding RNAs and their regulatory networks involved in Apis mellifera ligustica response to Nosema ceranae infection. BioRxiv (2019) DOI:**https://doi.org/10.1101/643627**[1]*.

**Table:** 

**Value of the data** • This transcriptome datasets provide comprehensive information about mRNAs, long non-coding RNAs and circular RNAs in normal and *N. ceranae-*stressed *A. m. ligustica* workers. • Our data offers novel insights into understanding interactions between *A. m. ligustica* and *N. ceranae.* • This data is useful for understanding the non-coding RNA-mediated mechanisms underlying the response of *A. m. ligustica* to *N. ceranae.*

## 1 Data

**Fig. 1:**
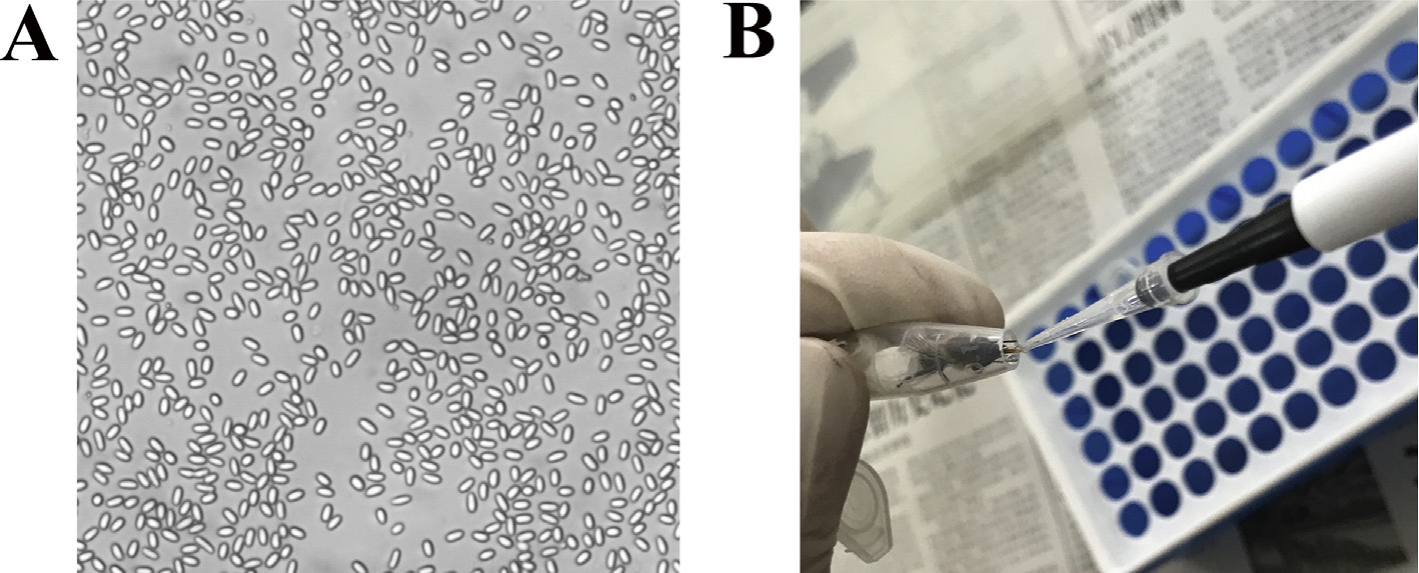
Artificial inoculation of *A. m. ligustica* worker with purified *N. ceranae* spores. A: Microscopic observation of purified *N. ceranae*spores (400 times magnification). B: Artificial inoculation of *A. m. ligustica* worker.

**Fig. 2:**
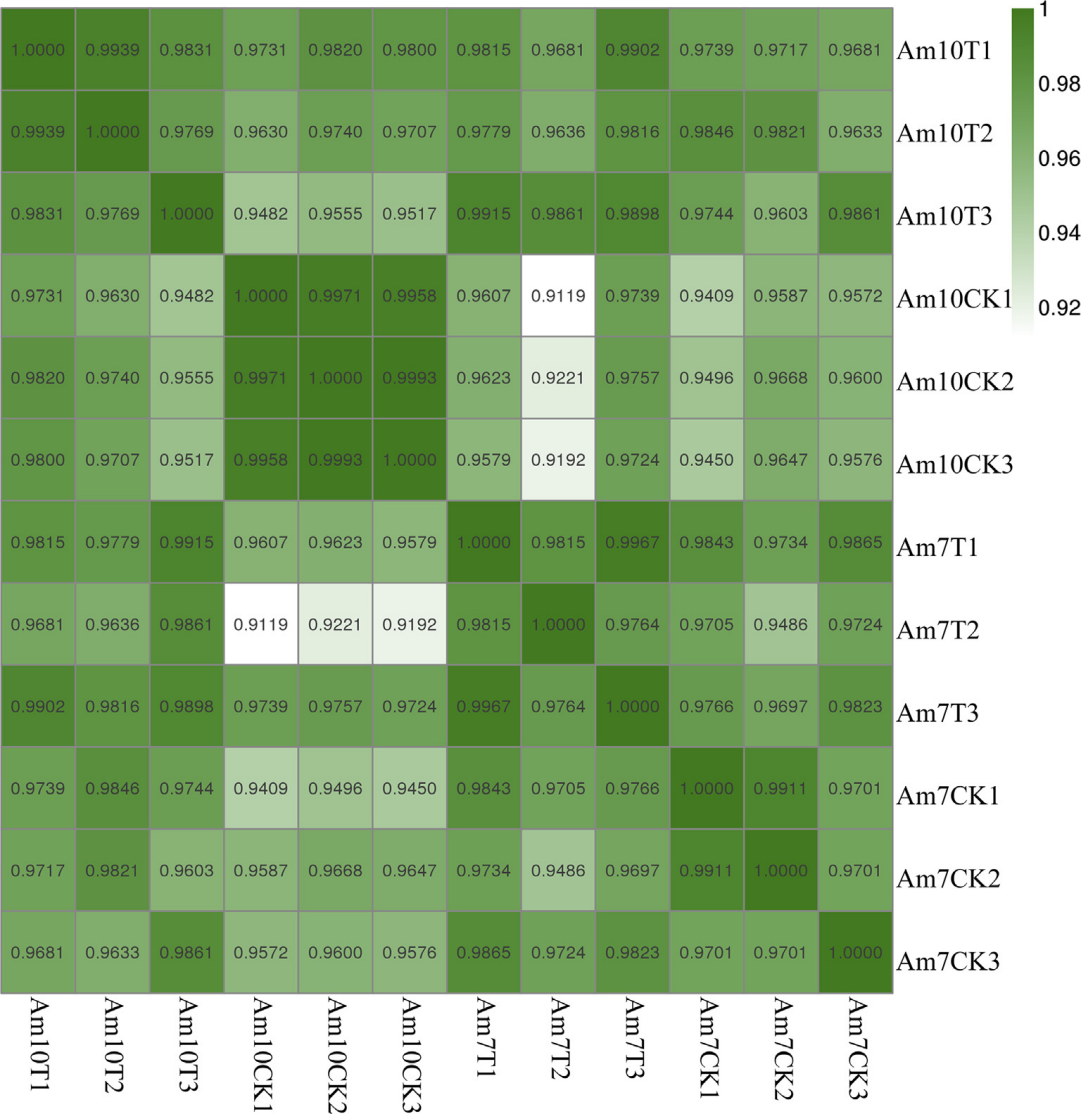
Pearson correlation coefficients between different repliacas within each *N. ceranae*-treated and control group.

*N. ceranae* spores (Fig. 1A) [1] were purified using Percoll discontinuous density gradient centrifugation. As Fig. 1B [1] shown, *A. m. ligustica* workers were starved for 2 h and then each artificially inoculated with 50% sucrose solution containing *N. ceranae* spores. The shared transcriptome data are from deep sequencing of untreated and *N. ceranae*-treated midguts of *A. m. ligustica* workers. On average, more than 23.32 Gb raw reads were gained from each group (Table 1) [1]. The quality control of transcriptome data shows clean reads in each group are over 99.42% (Table 1) [1]; in addition, Q20 (quality score is 20) and Q30 (quality score is 30) of clean reads in each group are above 96.98% and 93.34%, respectively (Table 1) [1]. Moreover, Pearson correlation coefficients of different biological replicas in each group are more than 91.19% (Fig. 2) [1]. The raw data were deposited in the Sequence Read Archive (SRA) database (http://www.ncbi.nlm.nih.gov/sra/) and connected to BioProject PRJNA406998.

**Table 1:** Quality control of transcriptome data.

Sample	Raw reads	Clean reads (%)	Q20 (%)	Q30 (%)
Am7CK1	160844082	160049106 (99.51%)	23340144349 (97.41%)	22521956996 (94.00%)
Am7CK2	129878194	129283918 (99.54%)	18891245674 (97.56%)	18239412915 (94.19%)
Am7CK3	113683898	113165446 (99.54%)	16535666991 (97.52%)	15943589998 (94.03%)
Am7T1	152323278	151668484 (99.57%)	22161043664 (97.55%)	21387125499 (94.15%)
Am7T2	200417896	199313090 (99.45%)	28948504448 (97.11%)	27829913730 (93.35%)
Am7T3	126667596	126053962 (99.52%)	18386919122 (97.38%)	17719616862 (93.85%)
Am10CK1	160537248	159765346 (99.52%)	23262715888 (97.27%)	22443038732 (93.84%)
Am10CK2	149230808	148494716 (99.51%)	21633348548 (97.28%)	20852891752 (93.77%)
Am10CK3	131386354	130619802 (99.42%)	18959297638 (96.98%)	18248516385 (93.34%)
Am10T1	249473666	248333982 (99.54%)	36162922479 (97.32%)	34857597435 (93.81%)
Am10T2	208589832	207574770 (99.51%)	30251988213 (97.34%)	29139831253 (93.77%)
Am10T3	173097006	172166682 (99.46%)	25113348781 (97.38%)	24175449594 (93.74%)

## 2 Experimental design, materials, and methods

### 2.1 *N. ceranae* spore purification

Fresh spores were isolated from naturally-infected foragers from a colony located at Fuzhou city, Fujian province, China, following the method described by Cornman et al. [2] with some minor modifications [3]. Firstly, bees were kept in −20 °C for 5 min followed by separation of midguts with clean dissection tweezers, homogenization in distilled water, filtration in four layers of sterile gauze, and then three times of centrifugation at 8000 rpm for 5 min at −4 °C; secondly, the supernatant was discarded as the spores remained in the sediment, the re-suspended pellet was further purified on a discontinuous Percoll gradient (Solarbio, China) consisting of 5 mL each of 25%, 50%, 75% and 100% Percoll solution, the spore suspension was overlaid onto the gradient and centrifuged at 14000 rpm for 90 min at 4 °C; thirdly, the spore pellet was carefully extracted with a sterile syringe followed by centrifugation again on a discontinuous Percoll gradient to gain clean spores (Fig. 1A) [1], which were immediately frozen in liquid nitrogen and stored at −80 °C until deep sequencing; fourthly, a portion of spores were subjected to PCR identification and proved to be mono-specific using previously described primers [4]; finally, the spore concentration was determined by counting using a CL kurt counter (JIMBIO, China). The spore suspension was freshly prepared before use.

### 2.2 Experimental design and sample collection

Frames of sealed brood obtained from a healthy colony of *A. m. ligustica* located in the teaching apiary of College of Bee Science, Fujian Agriculture and Forestry University were kept in an incubator at 34 ± 0.5 °C, 50% RH to provide newly emerged *Nosema*-free honeybees. The emergent workers were carefully removed, confined to cages in groups of 20, and kept in the incubator at 32 ± 0.5 °C, 50% RH. The bees were fed *ad libitum* with a solution of sucrose (50% w/w in water). One day after eclosion, the honeybees were starved for 2 h and 20 workers per group were each immobilized and then fed with 5 μL of 50% sucrose solution containing 1 × 10^6^ spores of *N. ceranae*, as shown in Fig. 1B [1]. Those individuals that did not consume the total amount of solution were discarded from the assay. After feeding, bees were isolated for 30 min in individual vials in the growth chamber to ensure that the sugar solution was not transferred among honeybees and the entire dosage was ingested. Control bees were inoculated in an identical manner using a 50% sucrose solution (w/w in water) without *N. ceranae* spores. Three replicate cages of 20 honeybees each were used in *N. ceranae*-treated and control groups. Each cage was checked every 24 h and any dead bees removed. *N. ceranae*-treated and control workers' midguts were respectively harvested at 7 d or 10 d post inoculation (dpi), immediately frozen in liquid nitrogen and kept at −80 °C until deep sequencing. *N. ceranae*-treated groups at 7 dpi and 10 dpi with sucrose solution containing *N. ceranae* spores were termed as Am7T (Am7T-1, Am7T-2, Am7T-3) and Am10T (Am10T-1, Am10T-2, Am10T-3); control groups at 7 dpi and 10 dpi with sucrose solution without *N. ceranae* spores were termed as Am7CK (Am7CK-1, Am7CK-2, Am7CK-3) and Am10CK (Am10CK-1, Am10CK −2, Am10CK-3).

### 2.3 RNA extraction, strand-specific cDNA library construction and deep sequencing

Total RNA of the six midgut samples from *N. ceranae*-treated groups and six midgut samples from control groups were respectively extracted using Trizol (Life Technologies) according to the manufacturer's instructions, and examined via 1% agarose gel eletrophoresis. Next, rRNAs were removed to retain mRNAs and ncRNAs, which were fragmented into short fragments by fragmentation buffer (Illumina) and reverse transcription into cDNA with random primers. Second-strand cDNA were synthesized by DNA polymerase I, RNase H, dNTP (dUTP instead of dTTP), and buffer. The cDNA fragments were purified using QiaQuick PCR extraction kit (QIAGEN, Germany), end repaired, poly(A) added, and ligated to Illumina sequencing adapters. UNG (Uracil-N-Glycosylase) (Illumina, USA) was then used to digest the second-strand cDNA. Ultimately, the digested products were size selected by agarose gel electrophoresis, PCR amplified, and sequenced on Illumina HiSeq™ 4000 platform (Illumina, USA) by Gene Denovo Biotechnology Co. (Guangzhou, China). All RNA sequencing data produced in our study are available in NCBI SRA database and connected to BioProject PRJNA406998.

### 2.4 Quality control and mapping of reads

Since reads produced from the sequencing machines included raw reads containing adapters or low quality bases which would affect the following assembly and analysis, raw reads were further filtered by removing reads containing adapters, more than 10% of unknown nucleotides (N), and more than 50% of low quality (*Q*-value ≤ 20) bases to obtain high quality clean reads. Quality control of transcriptome data is presented in Table 1 [1].

Short reads alignment tool Bowtie2 [5] was used for mapping reads to ribosome RNA (rRNA) database. The mapped reads were removed and the remaining reads were used in assembly and further analysis. The rRNA removed reads of each sample were then mapped to reference genome of *Apis mellifera* (assembly Amel_4.5) using TopHat2 (version 2.0.3.12) [6] following alignment parameters: (1) maximum read mismatch is two; (2) the distance between mate-pair reads is 50 bp; (3) the error of distance between mate-pair reads is ±80 bp.

### 2.5 Transcripts assembly

Transcripts were assembled using software Cufflinks [7], which together with TopHat2, allow researchers to identify novel genes and novel splice variants of known ones. The program reference annotation based transcripts (RABT) was preferred. Cufflinks constructed faux reads according to reference to make up for the influence of low coverage sequencing. During the last step of assembly, all of the reassembles fragments were aligned with reference genes and then similar fragments were removed. Cuffmerge was used to merge transcripts from different replicas of a group into a comprehensive set of transcripts, and the transcripts from multiple groups were then merged into a finally set of transcripts. Pearson correlations between every biological replicas in each sample group were calculated and shown in Fig. 2 [1].
